# Comparing shear wave elastography of breast tumors and axillary nodes in the axillary assessment after neoadjuvant chemotherapy in patients with node-positive breast cancer

**DOI:** 10.1007/s11547-024-01848-1

**Published:** 2024-07-26

**Authors:** Jia-Xin Huang, Feng-Tao Liu, Lu Sun, Chao Ma, Jia Fu, Xue-Yan Wang, Gui-Ling Huang, Yu-Ting Zhang, Xiao-Qing Pei

**Affiliations:** 1 Department of Medical Ultrasound, State Key Laboratory of Oncology in South China, Guangdong Provincial Clinical Research Center for Cancer, Sun Yat-Sen University Cancer Center, Guangzhou, 510060 China; 2 Breast Tumor Center, Sun Yat-Sen Memorial Hospital, Sun Yat-Sen University, Guangzhou, 510000 China; 3 Department of Pathology, Guangdong Provincial People’s Hospital (Guangdong Academy of Medical Sciences), Southern Medical University, Guangzhou, 510080 China; 4 Department of Pathology, State Key Laboratory of Oncology in South China, Guangdong Provincial Clinical Research Center for Cancer, Sun Yat-Sen University Cancer Center, Guangzhou, 510060 China; 5 Department of Liver Surgery, State Key Laboratory of Oncology in South China, Guangdong Provincial Clinical Research Center for Cancer, Sun Yat-sen University Cancer Center, Guangzhou, 510060 China

**Keywords:** Breast cancer, Elastography, Lymph node, Neoadjuvant chemotherapy

## Abstract

**Background:**

Accurately identifying patients with axillary pathologic complete response (pCR) after neoadjuvant chemotherapy (NAC) in breast cancer patients remains challenging.

**Purpose:**

To compare the feasibility of shear wave elastography (SWE) performed on breast tumors and axillary lymph nodes (LNs) in predicting the axillary status after NAC.

**Materials and Methods:**

This prospective study included a total of 319 breast cancer patients with biopsy-proven positive node who received NAC followed by axillary lymph node dissection from 2019 to 2022. The correlations between shear wave velocity (SWV) and pathologic characteristics were analyzed separately for both breast tumors and LNs after NAC. We compared the performance of SWV between breast tumors and LNs in predicting the axillary status after NAC. Additionally, we evaluated the performance of the most significantly correlated pathologic characteristic in breast tumors and LNs to investigate the pathologic evidence supporting the use of breast or axilla SWE.

**Results:**

Axillary pCR was achieved in 51.41% of patients with node-positive breast cancer. In breast tumors, there is a stronger correlation between SWV and collagen volume fraction (CVF) (*r* = 0.52, *p* < 0.001) compared to tumor cell density (TCD) (*r* = 0.37,* p* < 0.001). In axillary LNs, SWV was weakly correlated with CVF (*r* = 0.31, *p* = 0.177) and TCD (*r* = 0.29, *p* = 0.213). No significant correlation was found between SWV and necrosis proportion in breast tumors or axillary LNs. The predictive performances of both SWV and CVF for axillary pCR were found to be superior in breast tumors (AUC = 0.87 and 0.85, respectively) compared to axillary LNs (AUC = 0.70 and 0.74, respectively).

**Conclusion:**

SWE has the ability to characterize the extracellular matrix, and serves as a promising modality for evaluating axillary LNs after NAC. Notably, breast SWE outperform axilla SWE in determining the axillary status in breast cancer patients after NAC.

## Background

Neoadjuvant chemotherapy (NAC) followed by surgery is considered as the standard treatment for breast cancer patients with pathologically confirmed metastatic lymph nodes (LNs) [1]. According to the guidelines of the National Comprehensive Cancer network, nodal downstaging is the potential purpose of NAC for breast cancer with positive LNs. NAC has demonstrated the ability to eradicate axillary LN metastasis in approximately 40–50% of breast cancer patients [2]. However, accurately identifying patients with pathologic complete response (pCR) in axillary LNs remains challenging, and there is currently no consensus regarding the selection of appropriate candidates for less-invasive management. Consequently, axillary lymph node dissection (ALND) continues to be the reference standard for breast cancer patients with initially node-positive disease [3].

According to the guidelines of the American College of Radiology (ACR), ultrasound (US) is the preferred imaging modality for evaluating residual disease in the axillary LNs after NAC among breast cancer patients [4]. However, in node-positive breast cancer patients at pre-treatment, axillary US exhibits poor diagnostic performance in determining the status of LNs following NAC [5]. Ultrasound elastography (UE), an emerging technique in the field of US, has the potential to provide additional information about tissue stiffness. This stiffness is known to be associated with tumorigenesis and the progression of diseases [6]. UE appears to hold promise in distinguishing between malignant and benign breast lesions. Its potential in this regard has led to its inclusion in the 5th edition of the American College of Radiology breast imaging reporting and data system (ACR BI-RADS) lexicon [7, 8]. In addition, several studies have proved that the stiffness of breast tumors [9] or axillary LNs [10] prior to treatment can serve as a predictor of axillary status. And there is limited research regarding the role of UE in the assessment of axillary LNs following NAC in breast cancer patients. Huang JX et al. reported that SWE is a more accurate modality compared to conventional US for assessing axillary LNs after NAC [11]. However, there is no conclusive consensus regarding, whether UE should be performed on breast tumors or axillary LNs for evaluating the axillary region. Additionally, there is still a lack of histopathological explanation supporting the diagnosis of axillary LNs using UE after NAC. In order to provide compelling evidence for the clinical utility of UE techniques in axillary assessment after NAC, our study was designed to compare the values of breast and axilla UE in evaluating the axilla after NAC in patients with node-positive breast cancer, and also to explore the histopathological evidence supporting the use of breast or axilla UE in diagnosing axillary LNs after NAC.

## Methods

### Patients

**Fig. 1:**
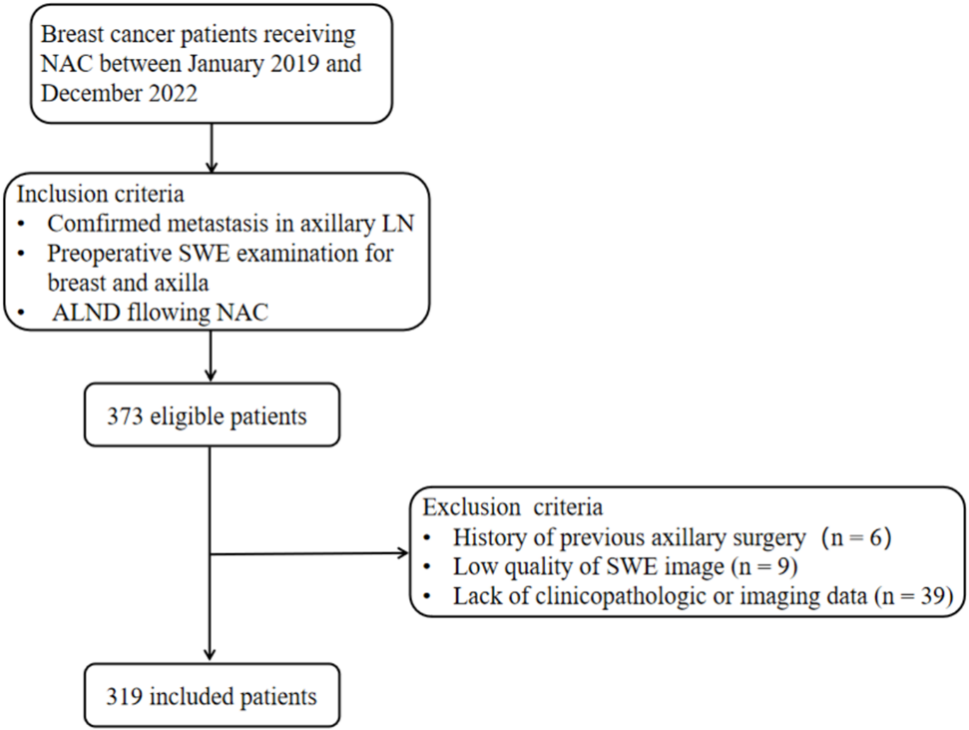
Flowchart of the study population. NAC, neoadjuvant chemotherapy; LN, lymph node; ALND, axillary lymph node dissection; SWE, shear wave elastography

In this prospective study, a total of 373 patients with breast cancer who received NAC were recruited between January 2019 and December 2022. The inclusion criteria of this study were as follows: (a) axillary LN metastasis was confirmed through core needle biopsy prior to treatment, (b) preoperative US examination was conducted for breast and axilla, and (c) underwent ALND after NAC. The exclusion criteria of this study were as follows: (a) a history of previous axillary surgery, (b) low quality of SWE image, and (c) lack of clinicopathologic or imaging data. As a result, a total of 319 patients were included in this study, as shown in Fig. 1. This study was approved by the ethics committee of the institutional review board (B2022-373-01). Written informed consent for study participation was obtained from all patients.

### US examination

**Fig. 2:**
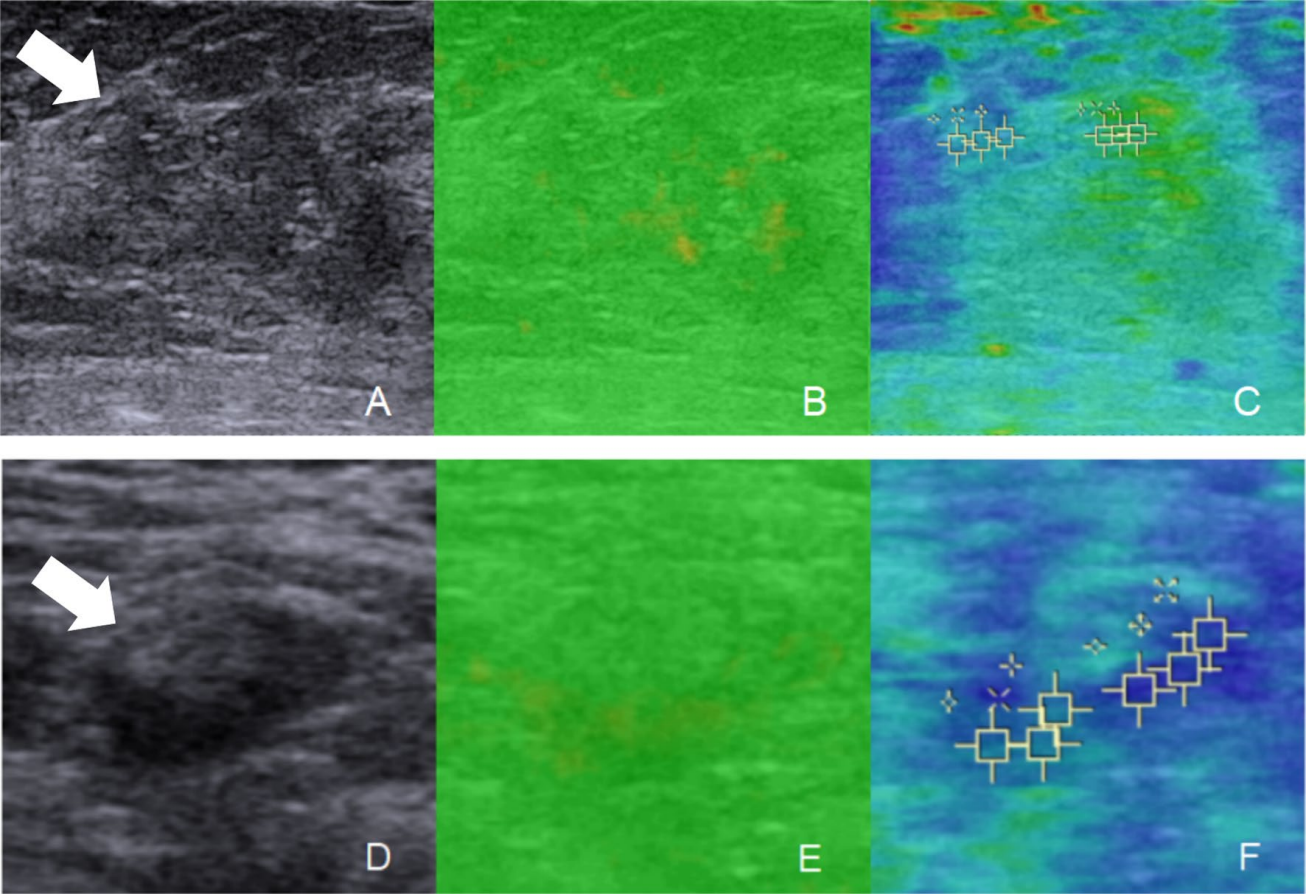
US examination of breast lesion and axillary LN in breast cancer patients after NAC. **A**, BUS image of the breast lesion (arrow); **B**, SWE quality map of the breast lesion (arrow); **C**, SWE velocity map and the corresponding SWV measurement of the breast lesion; **D**, BUS images of the axillary LN; **E**, SWE quality map of the axillary LN; **F**, SWE velocity map and SWV measurement of the axillary LN. The green areas on the quality map indicate high SW-quality, where SWV values were obtained. US, ultrasound; LN, lymph node; NAC, neoadjuvant chemotherapy; BUS, B-mode ultrasound; SWE, shear wave elastography, SWV, shear wave velocity

The US examination included conventional US and SWE using the Siemens Acuson S2000 ultrasound system (Siemens Medical Solutions, Mountain View, CA, USA) equipped with a 9 L4 linear array transducer. No markers, such as clips, were deployed in the breast tumors or axillary LNs before NAC. Breast cancer patients at our institute undergo regular re-evaluation through US after every two cycles of NAC [12]. One days before surgery, the patients underwent US examination for the breast and axilla. First, a conventional US scanning was performed to obtain B-mode US and color Doppler images of both breast tumors and axillary LNs in accordance with the BI-RADS lexicon. Second, SWE was performed on the largest section of residual breast lesions and axillary LNs. In the cases of multiple breast tumors, the evaluation was focused on the largest lesion. If no visible breast lesion was present, the region of the previous abnormality was considered as the target for assessment. The index for the axilla was defined as the LN with the most suspicious feature that appeared to have the maximum cortical thickness [11, 13]. SWE data were acquired, while the patients were instructed to momentarily suspend their respiration for approximately five seconds. During this time, the ultrasound probe was held steadily and lightly applied perpendicular to the skin surface. The SW-quality map is displayed in color mode to indicate the quality of SWE ranging from green (indicating high quality), to yellow (indicating intermediate quality), and to red (indicating low quality). The SW-velocity map is color-coded from blue to green or yellow to red to indicate different stiffness features, representing soft, intermediate, and hard areas. The shear wave velocity (SWV) values on the velocity map ranged from 0.5 to 10 m/s. SWV values were measured by placing three regions of interest (ROIs) measuring 2 × 2 mm over the areas with the highest and lowest stiffness in both the breast lesions and the cortex of LNs [14]. The six measured SWV values were averaged to calculate the mean SWV for further analysis [11, 15]. The results of US image acquisition and SWV measurement are shown in Fig. 2. To ensure repeatability, SWE examinations were performed three times for each case. All US images were obtained by one of three experienced radiologists who had been trained for at least six months according to the same imaging protocols. To assess the inter-observer agreement, three radiologists independently obtained the SWV values of the same breast lesions and axillary LNs in 30 cases.

### Pathological analysis

**Fig. 3:**
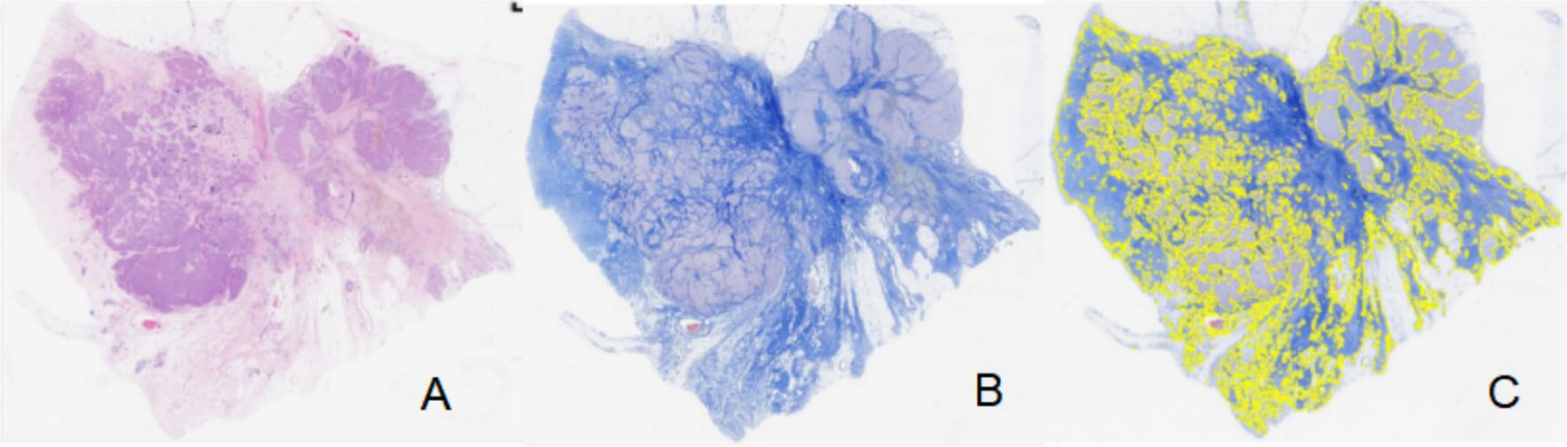
Example of CVF evaluation in WSI of a surgical specimen after NAC for breast cancer. **A** shows the WSI of the resected breast lesion stained with HE; **B** shows the corresponding WSI with Masson staining; **C** shows the area of WSI, where the collagen fiber component has been identified using the ImageJ software. CVF, collagen volume fraction; WSI, whole slide image; NAC, neoadjuvant chemotherapy; HE, hematoxylin and eosin

Invasive breast cancer and nodal metastasis were confirmed by US-guided core needle biopsy prior to treatment. After the completion of NAC, patients underwent mastectomy or breast-conserving surgery, accompanied by ALND. The surgical specimens were fixed in formaldehyde, embedded in paraffin, sliced into thin sections, and stained with hematoxylin and eosin (HE). Axillary pCR was defined as the absence of metastasis in all axillary LNs. Considering the significance of collagen as a key component of the extracellular matrix (ECM) and its involvement in breast cancer formation, invasion, and metastasis [16], the surgical specimens of breast lesions and axillary LNs were stained with Masson's trichrome staining [17]. This staining method was performed following the manufacturer's recommended protocol to assess the composition of collagen. Whole slide images (WSIs) of the surgical specimens were obtained using an automatic fluorescent slide scanner (Konfoong Biotech International, China) at a magnification of 40 times. The WSIs were then analyzed using the ImageJ software (https://imagej.net/software/imagej/) to determine the collagen volume fraction (CVF). Figure 3 illustrates the process of obtaining CVF from the WSIs. The WSIs of HE-stained sections were reviewed to determine tumor cell density (TCD) and necrosis proportion (NP). Regions with the highest TCD were identified, and three random fields were selected within these regions at a magnification of 40 × . Cell counting was performed in three 40 × fields per sample, and the results were converted to tumor cell numbers per square millimeter (count/mm^2^). The presence and extent of necrosis were visually assessed in the WSIs, and the ImageJ software was used to calculate the NP values.

### Statistical analysis

All statistical analyses were performed using SPSS 20.0 and Medical 16.2 software. Quantitative data were described using mean and standard deviation, while categorical variables were described using counts. The Spearman’s rank test was used to assess the inter-observer agreement of the SWE features. The SWV values with intraclass correlation coefficient (ICC) of > 0.75 were defined as reproducible imaging features [18]. A *t*-test or Mann–Witney *U* test was used to analyze the differences in quantitative data between the axillary pCR and residual metastasis groups. The *χ*2 test, Kruskal–Wallis test and Fisher exact test were employed to compare the categorical variables in this study. The correlations between SWV and pothologic characteristics were assessed using the Pearson correlation test or Spearman correlation test. Subsequently, the performances of SWV and CVF in predicting axillary status after NAC were assessed by receiver operating characteristic (ROC) curve analysis. Additionally, the Delong test was employed for pairwise comparison. A two-tailed *p* value of < 0.05 was considered statistically significant.

## Results

### Clinical and pathological data

A total of 319 patients with pathologically confirmed node-positive breast cancer who received NAC were included in this study. The median age of all patients was 45.53 ± 10.79 years, ranging from 27 to 70 years. All patients underwent ALND following NAC, with the number of cleared axillary lymph nodes ranging from 9 to 30. Among them, 164 cases (51.41%) achieved axillary pCR. Conversely, 155 cases (48.59%) were found to have residual metastasis in axillary LNs. The baseline clinical and pathological data of the enrolled cases are shown in Table 1. Significant differences were observed in age, clinical nodal stage, and molecular subtype between the axillary pCR and residual metastasis groups.

**Table 1:** Baseline characteristics of all patients

Characteristics	pCR (*n* = 164)	Residual metastasis(*n* = 155)	*p*-value
Age	42.58 ± 14.97	45.88 ± 14.61	0.047
Menopausal status			0.466
Pre/perimenopausal	108 (65.9)	96 (61.9)	
Postmenopausal	56 (34.1)	59 (38.1)	
Tumor stage			0.161
1	23 (14.0)	12 (7.7)	
2	92 (56.1)	83 (53.5)	
3	33 (20.1)	37 (23.9)	
4	16 (9.8)	23 (14.8)	
Nodal stage			0.001
1	64 (39.0)	39 (25.2)	
2	58 (35.4)	45 (29.0)	
3	42 (25.6)	71 (45.8)	
Clinical stage			0.129
2	47 (28.7)	33 (21.3)	
3	117 (71.3)	122 (78.7)	
Molecular subtypes			< 0.001
Luminal A	8 (4.9)	34 (21.9)	
Luminal B	102 (62.2)	79 (51.0)	
HER2 positive	33 (20.1)	18 (11.6)	
Triple-negative	21 (12.8)	24 (15.5)	

*HER2* human epidermal growth factor receptor2

### Inter-observer agreement of SWE

In the evaluation of breast SWE, the median ICC was found to be 0.82 (interquartile range: 0.77–0.90), while for axilla SWE evaluation, the median ICC was 0.80 (interquartile range: 0.76–0.86). These results indicate the high reproducibility in the acquisition and measurement of SWE data.

### Correlations between SWE and pathologic characteristics after NAC

This study analyzed the correlations between SWE characteristics and pathologic characteristics in both breast tumors and axillary LNs after NAC. In breast tumors, a significantly positive correlation was observed between SWV and CVF (*r* = 0.52, *p* < 0.001), indicating a strong and direct relationship between the two variables. Additionally, a significantly positive correlation was also observed between SWV and TCD in breast tumors (*r* = 0.37, *p* < 0.001), suggesting a moderate relationship between SWV and TCD. In axillary LNs, there was a positive correlation between SWV and CVF, although the correlation was weak (*r* = 0.31, *p* = 0.177). Similarly, SWV was weakly correlated with TCD (*r* = 0.29, *p* = 0.213). There is no significant relationship between SWV and NP in either breast lesions (*r* = 0.075, *p* = 0.358) or axillary LNs (*r* = 0.15, *p* = 0.304).

### Difference in SWE between axillary response groups

Table 2 shows the SWV measurements of breast lesions and axillary LNs after NAC. The SWV of breast tumors in all cases was 3.02 ± 1.34 m/s, which was significantly higher than that of axillary LNs (1.80 ± 0.49 m/s). Moreover, significant differences in SWV were observed between the two axillary response groups, both for breast lesions and axillary LNs (*p* < 0.01). Notably, the SWV of breast lesions in the axillary pCR group was 2.27 ± 0.86 m/s, lower than that in the axillary residual metastasis group (3.79 ± 1.31 m/s). Similarly, the SWV of LNs was 1.62 ± 0.28 m/s in the axillary pCR group and 1.98 ± 0.58 m/s in the axillary residual cancer group. Appendix Fig. 7 shows the comparison of SWE measurements for breast lesions and axiallary LNs between the axillary pCR and residual metastasis groups.

**Table 2:** SWE characteristics of both breast lesions and axillary LNs

SWV (m/s)	Total (*n* = 319)	Axillary pCR (*n* = 164)	Residual metastasis (*n* = 155)	*p*-value
Breast lesions	3.02 ± 1.34	2.27 ± 0.86	3.79 ± 1.31	< 0.001
Axillary LNs	1.80 ± 0.49	1.62 ± 0.28	1.98 ± 0.58	< 0.001

*SWE* shear wave elastography, *LN* lymph node, *SWV* shear wave velocity, *pCR* pathological complete response

### Difference in pathologic characteristics between axillary response groups

Table 3 shows the CVF values of breast lesions and axillary LNs in breast cancer patients after NAC. The CVF of breast tumors was found to be 40.47 ± 18.13%, which was significantly higher than that of axillary LNs (14.18 ± 10.53%). Consistent with the findings for SWV, the results revealed that the CVF value of breast lesions in axillary pCR group (30.11 ± 17.05%) was significantly lower than that in residual metastasis group (51.12 ± 12.05%). Similarly, a significant difference in the CVF of LNs was observed between the axillary pCR (10.37 ± 7.91%) and residual metastasis (18.10 ± 11.47%) groups. Table 4 presents the TCD values of breast lesions and axillary LNs after NAC. The TCD of breast tumors was found to be 1605 ± 2034 cells per mm^2^, which was significantly higher than that of axillary LNs (925 ± 2188 cells per mm^2^). And TCD value of breast lesions in axillary residual metastasis group (2217 ± 2130 cells per mm^2^) was significantly higher than that in pCR group (1026 ± 1734 cells per mm^2^). Table 5 presents the NP values of breast lesions and axillary LNs after NAC for breast cancer. In this study, necrotic tissue was identified in breast lesions in four cases and in axillary LNs in six cases. In the Appendix, Figure 8, Figure 9 and Figure 10 present the comparisons of the CVF, TCD and NP of breast lesions and axillary LNs between the two axillary response groups.

**Table 3:** ECM characteristics of both breast lesions and axillary LNs

CVF (%)	Total (*n* = 319)	Axillary pCR (*n* = 164)	Residual metastasis (*n* = 155)	*p*-value
Breast lesions	40.32 ± 18.13	30.11 ± 17.05	51.12 ± 12.05	< 0.001
Axillary LNs	14.13 ± 10.53	10.37 ± 7.91	18.10 ± 11.47	< 0.001

*ECM* extracellular matrix, *LN* lymph node, *CVF* collagen volume fraction, *pCR* pathological complete response

**Table 4:** Tumor cell characteristics of both breast lesions and axillary LNs

TCD (count/mm^2^)	Total (*n* = 319)	Axillary pCR (*n* = 164)	Residual metastasis (*n* = 155)	*p*-value
Breast lesions	1605 ± 2034	1026 ± 1734	2217 ± 2130	< 0.001
Axillary LNs	925 ± 2188	0 ± 0	1904 ± 2082	< 0.001

*LN* lymph node, *TCD* tumor cell density, *pCR* pathological complete response

**Table 5:** Necrosis characteristics of both breast lesions and axillary LNs

NP (%)	Total (*n* = 319)	Axillary pCR (*n* = 164)	Residual metastasis (*n* = 155)	*p*-value
Breast lesions	1.15 ± 8.44	0.23 ± 1.98	1.98 ± 11.45	0.184
Axillary LNs	2.42 ± 13.07	0 ± 0	4.60 ± 17.78	0.023

*LN* lymph node, *NP* necrosis proportion, *pCR* pathological complete response

### The performance of SWE and ECM characteristics for assessing axilla after NAC

**Fig. 4:**
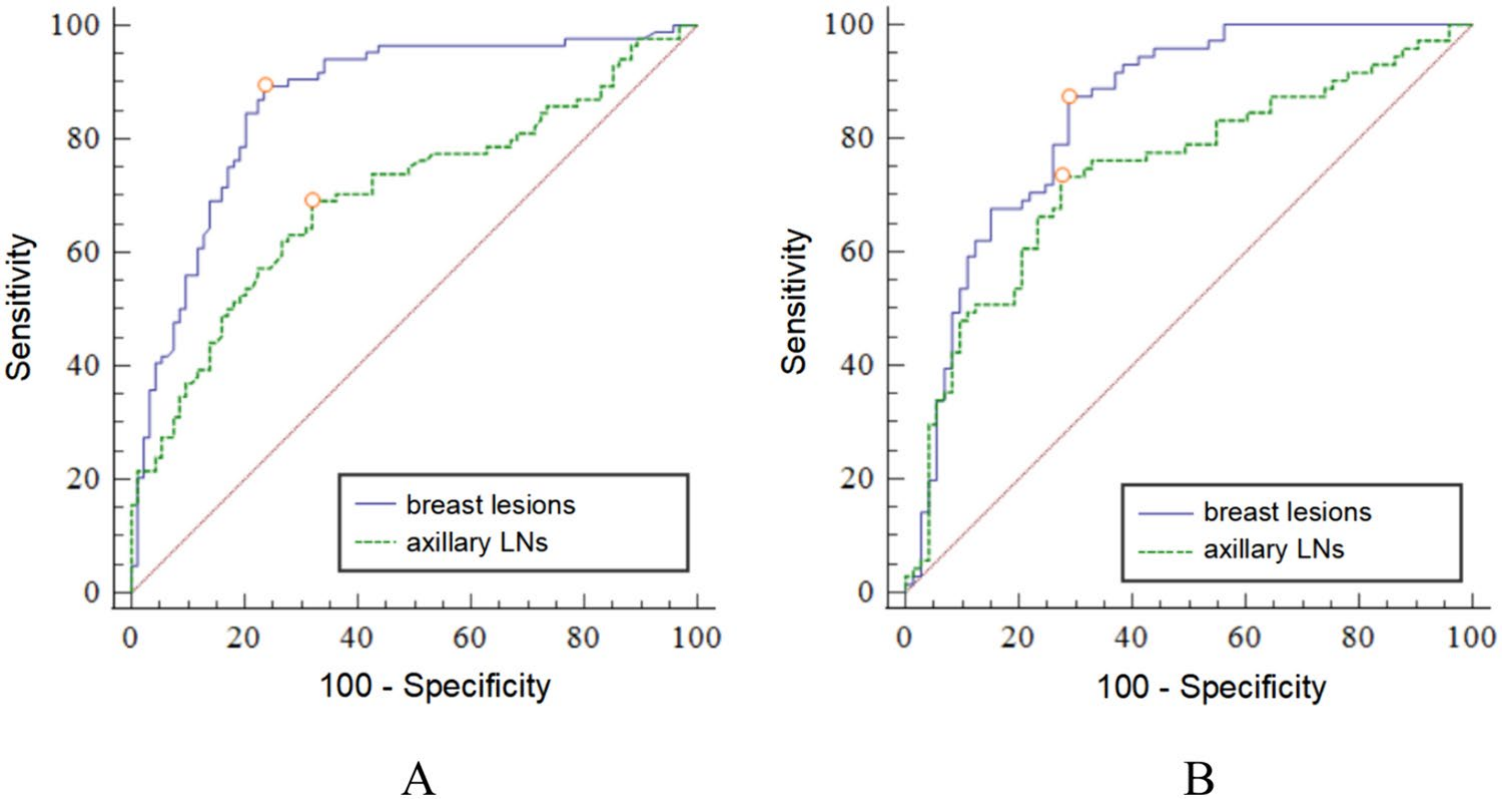
ROC curves for the SWV (**A**) and CVF (**B**) of breast lesions and axillary LNs in predicting axillary responses to NAC. ROC, receiver operating characteristic; SWV, shear wave velocity; CVF, collagen volume fraction; LN, lymph node; NAC, neoadjuvant chemotherapy

**Fig. 5:**
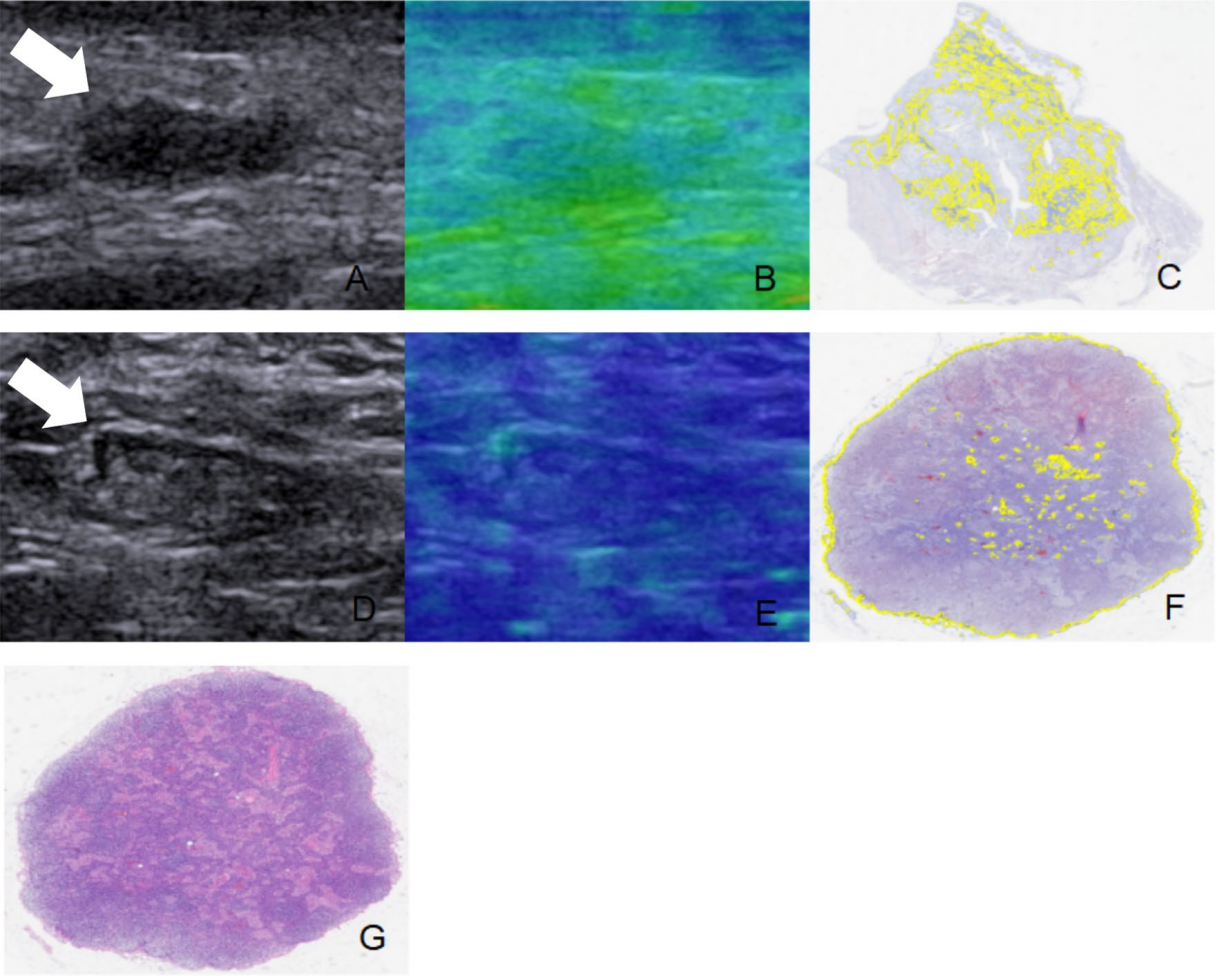
A breast cancer case with axillary pCR after NAC. A 32 year-old female patient with HER2 positive breast cancer had a residual breast lesion (arrow) with the size of 12 × 4 mm (**A**). The SWV of the breast lesion was measured to be 2.21 m/s, which was lower than the threshold value of 2.47 m/s (**B**). In addition, the CVF of the breast lesion was found to be 17.44%, which was lower than threshold value of 37.67% (**C**). For the LN (arrow) that was examined by US following NAC, it had a size of 11 × 4 mm, an oval shape, and a cortical thickness of 1.1 mm (**D**). The SWV of the LN was measured to be 1.33 m/s (**E**), and the CVF was 3.7% (**F**). A total of 16 axillary LN were resected after NAC, and no metastasis was found in all LNs (**G**). pCR, pathological complete response; NAC, neoadjuvant chemotherapy; HER2, human epidermal growth factor receptor2; SWV, shear wave velocity; CVF, collagen volume fraction; LN, lymph node; US, ultrasound

**Fig. 6:**
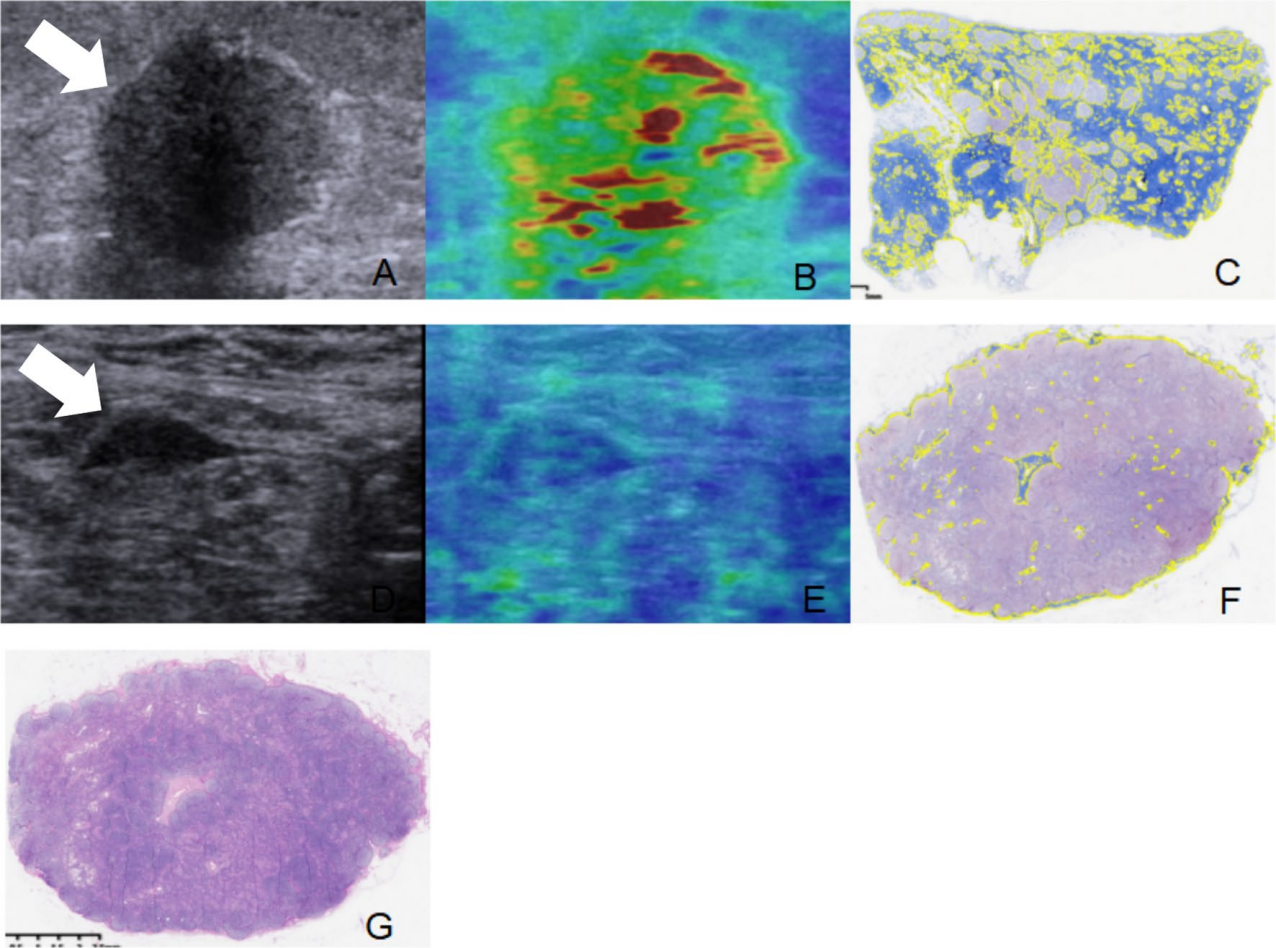
A breast cancer case with residual metastasis in axillary LNs after NAC. The patient was a 36 year-old female with Luminal B breast cancer who received eight courses of NAC. After the completion of NAC, a residual breast lesion (arrow) with a size of 18 × 13 mm was observed (**A**). The SWV of the breast lesion was measured to be 5.36 m/s (**B**), which was higher than threshold value of 2.47 m/s. In addition, the CVF of the breast lesion was found to be 62.88% (**C**), exceeding the threshold value of 37.67%. **D** shows a LN (arrow) with suspicious features on US. This LN exhibited a size of 26 × 8 mm, an outwards bulging shape, and a focal cortical thickening of 4.4 mm. The SWV of the LN was measured to be 1.48 m/s (**E**), and the CVF was 4.5% (**F**). Following NAC, a total of 19 axillary LNs were resected, and metastasis was found in two of them (**G**). LN, lymph node; NAC, neoadjuvant chemotherapy; SWV, shear wave velocity; CVF, collagen volume fraction; LN, lymph node; US, ultrasound

**Fig. 8:**
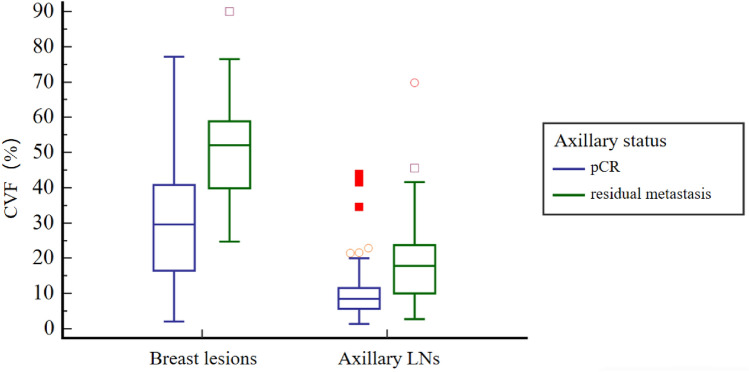
Clustered box plot of the CVF of breast lesions and axillary LNs in both nodal pCR and nodal residual disease groups. CVF, collagen volume fraction; LN, lymph node; pCR, pathological complete response

**Fig. 9:**
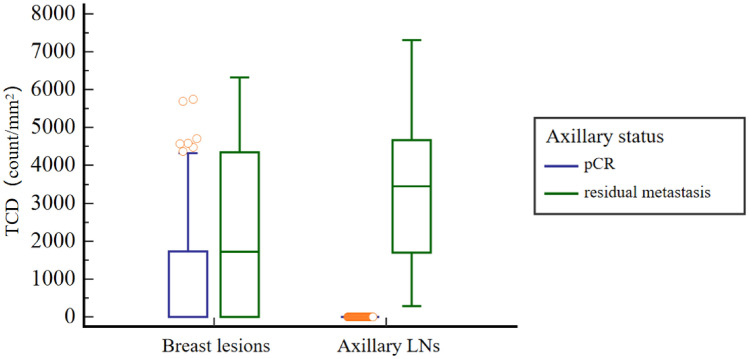
Clustered box plot of the TCD of breast lesions and axillary LNs in both nodal pCR and nodal residual disease groups. TCD, tumor cell density; LN, lymph node; pCR, pathological complete response

**Fig. 10:**
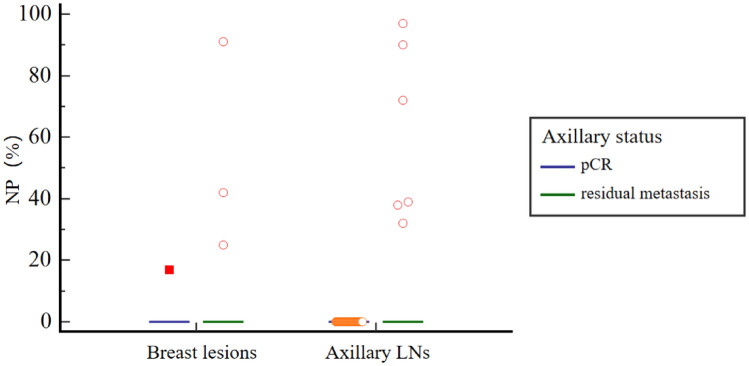
Clustered box plot of the NP of breast lesions and axillary LNs in both nodal pCR and nodal residual disease groups. NP, necrosis proportion; LN, lymph node; pCR, pathological complete response

Among the pathological characteristics evaluated in our study, we found that collagen deposition played a crucial role in contributing to tissue stiffness after NAC in breast cancer patients. To compare the efficacy of breast and axilla SWE in assessing the axillary response to NAC and to explore the pathological evidence supporting the use of breast or axilla SWE in this context, Table 6 provides a summary of the performances of both SWV and CVF in evaluating the axillary status after NAC. The AUCs for SWV were 0.87 and 0.70 in breast lesions and axillary LNs, respectively. The Delong test demonstrated that SWV was significantly greater in breast lesions than in axillary LNs for predicting the axillary response to NAC (*p* < 0.001), as shown in Fig. 4A. Similarly, CVF in breast lesions (AUC = 0.85) performed better than that in axillary LNs (AUC = 0.74) for predicting the axillary response (Delong test, *p* = 0.048), as shown in Fig. 4B. For SWV in breast lesions, the ACC, SEN, SPE, PPV and NPV were 82.76%, 88.39%, 77.44%, 78.74% and 87.59%, respectively, with a threshold value of 2.47 m/s. The SWV in axillary LNs showed a moderate performance with a ACC of 68.65%, SEN of 69.03%, SPE of 68.29%, PPV of 67.30%, NPV of 70.00%, and SWV threshold of 1.66 m/s. Figures 5 and 6 illustrate the US and pathological analysis of breast lesions and axillary LNs in breast cancer patients with axillary pCR and residual metastasis after NAC.

**Table 6:** The performances of SWV and CVF in predicting the axillary response to NAC

Characteristics	AUC	ACC (%)	SEN (%)	SPE (%)	PPV (%)	NPV (%)	YI	Cu-off value
SWV_breast_ (m/s)	0.87	82.76 264/319	88.39 137/155	77.44 127/164	78.74 137/174	87.59 127/145	0.66	2.47
SWV_LN_ (m/s)	0.70	68.65 219/319	69.03 107/155	68.29 112/164	67.30 107/159	70.00 112/160	0.37	1.66
CVF_breast_ (%)	0.85	79.62 254/319	87.74 136/155	71.95 118/164	74.73 136/182	86.13 118/137	0.60	37.67
CVF_LN_ (%)	0.74	72.73 232/319	72.90 113/155	72.56 119/164	71.52 113/158	73.91 119/161	0.45	10.58

*SWV* shear wave velocity, *CVF* collagen volume fraction, *NAC* neoadjuvant chemotherapy, *AUC* area under receiver operating characteristic curve, *ACC* accuracy, *SEN* sensitivity, *SPE* specificity, *PPV* positive predictive value, *NPV* negative predictive value, *YI* youden index, *LN* lymph node

## Discussion

This study suggests that SWE can characterize ECM in the tumor microenvironment and serve as a promising modality for evaluating axillary LNs after NAC. The performance of SWE on breast tumors is more effective than that on axillary LNs for determining the axilla status in patients with initially node-positive breast cancer after NAC.

In our study, NAC successfully eradicated nodal disease in 51.41% of the patients included in our analysis. However, accurately identifying patients who are likely to achieve a nodal pCR after NAC remains a challenge. As the most recommended imaging modality for the assessment of residual disease in axillary LNs [4], the accuracy of conventional US in the evaluation of axillary LNs after NAC is insufficient, and there is a great variability in its diagnostic performance [19–22]. And several studies have reported that UE can improve the performance of US in diagnosing axillary LNs [9, 23]. In a meta-analysis evaluating eight imaging modalities for the detection of metastasis in axillary LNs in breast cancer patients, UE was found to be the most effective method for preoperative detection [23]. In the context of assessing axillary status after NAC, only a few studies have focused on the role of UE [12]. And the pathological basis for using UE to evaluate axillary LNs after NAC is unclear, particularly regarding the preferred approach for assessing axillary status—whether UE should be performed on breast tumors or axillary LNs.

In our study, there were stronger positive correlations between SWV and CVF compared to TCD for both breast tumors and axillary LNs after NAC. Similarly, Chamming's F reported that there was a very significant correlation between elasticity and fibrosis in breast cancer prior to treatment, but no significant correlation with viable cellular tissue [17]. These findings suggest that collagen deposition may contribute more to tumor stiffness than tumor cell presence. In the context of breast lesions, the presence of necrotic tissue can indicate advanced disease or aggressive tumor behavior. Similarly, the presence of necrotic tissue in axillary LNs can suggest advanced disease and may be associated with the spread of malignant tumors to the LNs. Based on our study findings, however, necrosis is rarely observed in breast lesions or axillary LNs after NAC. This implies that the presence of necrosis has little impact on the detection of residual metastasis in axillary LNs. The results of this study concluded that collagen deposition was identified as the primary factor contributing to tissue stiffness in breast cancer patients after NAC.

This study demonstrated that the CVF of breast tumors in axillary residual metastasis group was found to be significantly higher than that in axillary pCR group. These findings indicate the presence of a greater cancer-associated collagen composition in breast tumors with positive LNs after NAC. Correspondingly, this study found that the breast lesions in axillary residual metastasis group exhibited higher stiffness compared to those in axillary pCR group. This study suggests that SWE has the potential to evaluate ECM characteristics in breast cancer after NAC, and the breast SWE can reflect the differences in collagen deposition within breast tumors between patients with negative and positive LNs. Abnormal collagen composition within the tumor microenviroment can contribute to increased interstitial pressure, which in turn can lead to the collapse of tumor vessels [24, 25]. This collapse reduces tumor perfusion, thus limiting the delivery of chemotherapy drugs to the tumor site. Consequently, tumors with higher stiffness, as indicated by SWE, may indicate resistance to chemotherapy. This study further proves that breast SWE can characterize the collagen composition within breast tumors after NAC and have the potential to act as predictors for axillary responses to NAC.

Similar to breast tumors, this study found that the SWV values of axillary LNs in axillary residual metastasis group were significantly higher than those in axillary pCR group. The presence of metastatic foci within LNs can lead to an increase in their stiffness due to various factors, including the increased of matrix and cytoskeletal stiffness, abnormal cell proliferation, microcalcification of malignant lesions, and deposition of other abnormal tissues in the stroma [26]. Several studies have demonstrated that axilla SWE can be used to evaluate metastasis in LNs both in vivo [27] and in vitro [28]. In this study, we found that the SWE performed on axillary LNs were not as effective as that performed on breast tumors for the detection of axillary residual metastasis after NAC. As the pathological characteristic contributing most to stiffness after NAC, collagen composition in axillary LNs is also less effective than in breast tumors for assessing axillary status. Pathological analysis revealed that the difference in CVF of axillary LNs was smaller compared to that of breast tumors when distinguishing between the positive and negative LN groups after NAC. Similarly, a smaller difference in the SWV of axillary LNs than breast tumors was also observed between the two axillary response groups. This implies that axilla SWE carries a higher risk in false negative and false positive results than breast SWE for evaluating axillary LNs.

Upon retrospective review of false negative cases, there was only minimal collagen deposition in axillary LNs, even in those with residual metastasis. Unlike primary breast malignancies, metastases in axillary LNs may rarely induce a desmoplastic reaction [11]. Thus, we speculate that axilla UE may lead to underestimation in the diagnosis of axillary LNs. In addition, we observed that LNs in a significant portion of false positive cases were located in a deep position (vertical distance from epidermis > 2.5 cm). It has been suggested that the perpendicular depth of LN location may affect the signal stability of UE [29]. Therefore, it can be inferred that UE of LNs in deep axillary location may lead to overestimation of elasticity, thereby resulting in false positive findings. In addition, compared to the breast, the potential impact of artifacts and a more complex acquisition process in the axillary region might also contribute to the lower performance of axilla SWE.

In the context of NAC for breast cancer, this study suggested that SWE can be utilized to assess collagen deposition in breast tumors and axillary LNs. These findings provided pathological evidence supporting the use of SWE for diagnosing axillary LNs after NAC in patients with node-positive breast cancer. More importantly, the study proved that breast SWE is the preferred approach for evaluating the axillary status after NAC, as it demonstrates inherent advantages over axilla SWE, as confirmed by pathological analysis. Nevertheless, this study has some limitations that should be acknowledged. First, this study is limited by its single-center design. Second, it is important to note that we did not conduct a comparison between SWE and the standard evaluation for LNs (conventional axilla US). Further research is needed to comprehensively assess the effectiveness of SWE in this context. Third, due to the absence of clip placement within biopsied LNs, it was challenging to determine the pathological status of index LNs before treatment. Further, it is known that NAC impacts tumor extracellular collagen in a complex and subtype-specific manner [30], but we did not conduct a subgroup analysis based on the specific molecular type of breast cancer in our study. Finally, there is no specific pairing process implemented to directly correlate the LNs observed using SWE with their corresponding pathology analysis. The axillary US assessment focused on the LNs with the most suspicious features, and it is likely that the pathologically analyzed LNs include those that have been identified and analyzed during the imaging process.

## Conclusion

In summary, SWE is a feasible imaging biomarker for evaluating pathological characteristics in both breast lesion and axillary LNs after NAC in patients with initially node-positive breast cancer. Compared to SWE performed directly on the axilla, breast SWE is a preferred approach for determining the axillary status after NAC.

## Figures and Tables

**Fig. 7:**
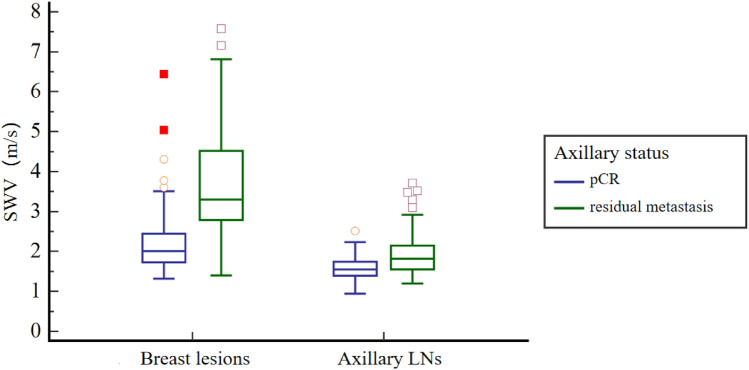
Clustered box plot of the SWV of breast lesions and axillary LNs in both nodal pCR and nodal residual disease groups. SWV, shear wave velocity; LN, lymph node; pCR, pathological complete response

## Data Availability

The authors declare that they had full access to all of the data in this study and the authors take complete responsibility for the integrity of the data and the accuracy of the data analysis.

## Appendix

See Figs. 7, 8, 9 and 10.

## Abbreviations

ALND: Axillary lymph node dissection
AUC: Area under the ROC
BUS: B-mode US
CVF: Collagen volume fraction
ECM: Extracellular matrix
HE: Hematoxylin and eosin
HER2: Human epidermal growth factor receptor2
LN: Lymph node
NAC: Neoadjuvant chemotherapy
NP: Necrosis proportion
pCR: Pathological complete response
ROC: Receiver operating characteristic
SWE: Shear wave elastography
SWV: Shear wave velocity
TCD: Tumor cell density
US: Ultrasound
UE: Ultrasound elastography
WSI: Whole slide image
